# Association between Paramedic Workforce and Survival Rate in Prehospital Advanced Life Support in Out-of-Hospital Cardiac Arrest Patients

**DOI:** 10.1155/2022/9991944

**Published:** 2022-03-17

**Authors:** Kichan Han, You Hwan Jo, Yu Jin Kim, Seung Min Park, Dong Keon Lee, Dong Won Kim, Kui Ja Lee, Hyo Ju Choi, Dong-Hyun Jang

**Affiliations:** ^1^Department of Emergency Medicine, Seoul National University Bundang Hospital, Seongnam 13620, Republic of Korea; ^2^Department of Emergency Medicine, Seoul National University College of Medicine, Seoul 03080, Republic of Korea; ^3^Department of Emergency Medicine, Chuncheon Sacred Heart Hospital, Hallym University College of Medicine, Chuncheon 24253, Republic of Korea; ^4^Department of Emergency Medical Services, Kyungdong University, Wonju 26495, Republic of Korea; ^5^Department of Emergency Medicine, Korea University Anam Hospital, Seoul 02841, Republic of Korea

## Abstract

The low survival rate of out-of-hospital cardiac arrest (OHCA) patients is a global public health challenge. We analyzed the relationship between the number of prehospital EMS personnel and survival admission, survival discharge, and good neurologic outcomes in OHCA patients. This was a retrospective observational study. Adult nontraumatic OHCA patients from January 1, 2015, to December 31, 2018, were included from 12 cities in the Gyeonggi province, a metropolitan area located in the suburbs of the capital of the Republic of Korea. By comparing the insufficient EMS team (four or five EMS personnel) and the sufficient EMS team (six EMS personnel), we showed the survival rate of each group. Using propensity score matching, we reduced the bias of the confounding variables. A total of 3,632 OHCA patients were included. After propensity score matching, survival to admission was higher in the sufficient EMS team than in the insufficient EMS team (odds ratio (OR): 1.38, 95% confidence interval (CI): 1.04–1.84, *P*=0.03). Survival-to-discharge was similar (OR: 1.70, CI: 1.20–2.40, *P*=0.03), but there was no significant outcome in good neurologic outcomes (OR: 0.88, CI: 0.57–1.36, *P*=0.58). Our findings suggest that a sufficient EMS team (six EMS personnel) could improve the survival admission and discharge of OHCA patients compared to an insufficient EMS team (four or five EMS personnel). However, there was no significant difference in neurologic outcomes according to the number of EMS personnel.

## 1. Introduction

Cardiac arrest is a life-threatening emergency, and the low survival rate of out-of-hospital cardiac arrest (OHCA) is a global public health challenge. The average global incidence among adults is 0.1%, with 55 OHCAs per 100,000 person-years. In addition, the survival-to-discharge rate is 8.6% and good neurologic recovery (cerebral performance category (CPC) 1 and 2) is 5.1% in the Republic of Korea [1, 2].

For the survival of OHCA patients, prehospital high-quality cardiopulmonary resuscitation (CPR) and rapid defibrillation are essential, and prehospital advanced life support (ALS) by trained paramedics on the scene is essential [1, 3, 4].

An adequate number of emergency medical services (EMS) personnel are required for prehospital ALS; however, there are no guidelines for EMS staff regarding this number and the number of dispatched paramedics for OHCA patients differs by region. Several studies have related to the optimal number of paramedics required for prehospital ALS; however, each study notes a different value [5–7].

The survival rate and neurologic outcomes of OHCA patients are influenced by several factors, including shockable rhythm, bystander CPR, and a witnessed arrest. Therefore, we conducted propensity score matching using these factors and aimed to compare the outcomes according to the number of EMS personnel in adult patients with OHCA.

## 2. Materials and Methods

### 2.1. Study Setting and Design

This study is a retrospective analysis of prospectively collected OHCA registry data from January 1, 2015, to December 31, 2018. Eligible patients were adults (aged 18 years or older) with OHCA treated prehospital ALS in 12 cities of Gyeonggi province, a metropolitan area located in the suburbs of the capital of the Republic of Korea. Patients with obvious death and nonmedical causes (trauma, poisoning, and asphyxia) were initially excluded. In addition, previously poor cerebral performance category (CPC) scores of 3-4 were also excluded.

The primary outcome was survival of the OHCA patients till hospital discharge, depending on the number of paramedics at the scene, and the secondary outcomes were survival till admission and good neurologic recovery (CPC score 1-2).

This study was approved by the Institutional Review Board of Seoul National University Bundang Hospital (approval number: B-2105/684-105).

### 2.2. EMS System in the Study Region

In the Republic of Korea, the EMS system established by the government is operated by the National Fire Department, and the Korean EMS provides basic-to-intermediate ambulance services. When the emergency medical dispatcher recognizes a cardiac arrest, the two nearest available ambulances are dispatched to the scene. Prehospital ALS is performed under the direct medical control of an emergency physician through video calls at the site. Each ambulance has a crew comprising two or three emergency medical technicians (EMT), including at least one level-1 EMT, who are allowed to insert an intravenous catheter and an advanced airway tube under an emergency physician's direct medical control [8, 9]. Other members include level-2 EMTs (known as EMT-Basics in the US) or nurses. In the Republic of Korea, an emergency physician must complete a formal training course for direct medical control and be certified to conduct ALS according to the latest AHA guidelines. Therefore, most OHCA victims are supported by prehospital ALS comprising four to six EMS personnel except in cases of obvious clinical signs of irreversible death (e.g., rigor mortis, dependent lividity, decapitation, transection, or decomposition) or existing valid evidence indicating that resuscitation is not desired or refusal of resuscitation by the guardian [10]. When a patient with suspected cardiac arrest was reported, the dispatched first ambulance team notified the emergency physician and performed basic life support (BLS). In the case of two paramedics, one immediately started chest compression and the other performed a defibrillation using an automated external defibrillator (AED) if necessary. After using the AED, chest compression was done in rotation and Ambu bagging was performed by the other. In the case of three paramedics, chest compression and Ambu bagging were performed by the two and the other used the AED. Chest compression was done in rotation after using the AED as well. When the followed ambulance team arrived, BLS was switched to ALS under the medical guidance. Two or three level-2 EMTs performed chest compression and Ambu bagging. One of the level-1 EMTs applied the manual defibrillator and checked rhythm and then performed defibrillation if necessary. The other level-1 EMT secured an intravenous line and administered the drug. If chest compression, defibrillation, and drug administration were done successfully, one of the level-1 EMTs inserted an advanced airway. Level-1 EMT from the guardian took the patient's medical history, after all these ALS went well. The manual chest compression was performed based on the guidelines on the scene. Some ambulances in each group used mechanical thumper compression devices during transport. The direct medical control was performed using a mobile phone and Bluetooth headset. BLS was performed instead of ALS in the absence of a video call.

A hospital transfer occurs when return of spontaneous circulation (ROSC) occurs or when the emergency physician determines that transfer to the hospital is preferable over continuing ALS on-site, taking into account the presumed cause of cardiac arrest or the patient's condition.

### 2.3. Data Collection and Processing

Data were collected through an EMS run sheet and the central cardiac arrest registry from the EMS providers. In addition, OHCA registries were collected for hospital care and survival outcomes using hospital electronic medical records. The following data were obtained: age, sex, witnessed arrest (a cardiac arrest that is seen or heard by another person or is monitored), bystander CPR (cardiopulmonary resuscitation performed by a person who was not a part of an organized emergency response system to the cardiac arrest), initial shockable rhythm (the first monitored cardiac rhythm was ventricular fibrillation or pulseless ventricular tachycardia), response time interval (RTI) (the time from EMS call to arrival at the scene), scene time interval (STI) (the time from ambulance arrival at the scene to departure to hospital), number of EMS team personnel dispatched to the scene, prehospital ROSC, survival to admission, survival to discharge, and neurological outcome at hospital discharge. The first monitored rhythm was classified as shockable, pulseless electrical activity (PEA), and asystole. The number of EMS team personnel from the EMS run sheet was defined and divided into a sufficient EMS team (six EMS personnel) and an insufficient EMS team (four to five EMS personnel). Prehospital ROSC was defined as the achievement of ROSC at any point before arriving at the hospital.

### 2.4. Computation and Matching of Propensity Score

Propensity scoring (PS) was used to reduce selection bias between a sufficient EMS team and an insufficient EMS team. Logistic regression analysis was used to calculate the PS based on patient characteristics, including age, sex, witnessed arrest, initial shockable rhythm, and bystander CPR. A PS analysis of 1 : 1 matching was performed without replacement, using the nearest neighbor method with a maximum caliper of 0.5 to generate matched pairs of patients.

### 2.5. Statistical Analysis

Continuous variables were compared using Student's *t*-test or Mann−Whitney U test, and categorical variables were compared using the *χ*^2^ test or Fisher's exact test. Statistical significance was set at *P* < 0.05.

Multivariable logistic regression was conducted to examine the association between the number of EMS team personnel and outcomes. Multivariable models included age, sex, witnessed arrest (or not), bystander CPR (or not), initial shockable rhythm (or not), and RTI and STI as covariates. Adjusted odds ratios (ORs) with 95% confidence intervals (CIs) were also calculated. A matching process based on the PS was used to equalize the potential prognostic factors in both groups.

Data were analyzed using the Statistical Package for the Social Sciences (SPSS) for Windows version 27.0 (SPSS Inc., Chicago, IL, USA). A two-sided *P* value <0.05 was considered statistically significant.

## 3. Results and Discussion

**Figure 1:**
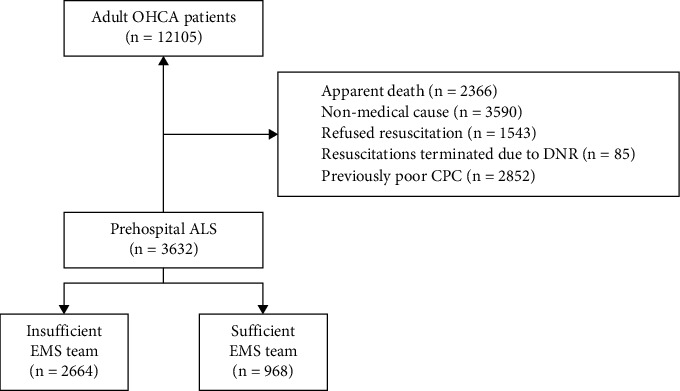
Flowchart showing study inclusion and exclusion criteria. OHCA, out-of-hospital cardiac arrest; DNR, do not resuscitate; CPC, cerebral performance category; ACLS, advanced cardiovascular life support; EMS, emergency medical services.

During the study period, a total of 12,105 OHCA patients underwent EMS activation for cardiac arrest. Among them, 3,632 patients were included, excluding 2,366 cases in which CPR was not performed because of obvious death, 3,590 cases with no medical cause, 1,543 cases of CPR rejection, 85 cases of CPR suspension due to do not resuscitate (DNR) instructions, and 2,852 cases of CPC 3 or 4 before cardiac arrest. Applying the inclusion criteria, 2,664 (73.3%) were insufficient EMS teams and 968 (26.7%) were sufficient EMS teams (Figure 1).

Table 1 displays the distribution of age, sex, and cardiac arrest factors according to the number of EMS team personnel before and after propensity score matching (PSM). The median ages of the insufficient EMS and sufficient EMS teams were 68 and 68.5 years, respectively. After PSM, all factors were similar, including mean age (66.0 years vs. 66.0 years), male (66.9% vs. 67.8%), witnessed arrest (39.5% vs. 39.8%), bystander CPR (67.5% vs. 67.1%), and initial shockable rhythm (19.7% vs. 21.1%) in the insufficient EMS team and sufficient EMS team.

Overall survival to admission and discharge was 27.2% and 15.3% in the sufficient EMS team group and 20.3% and 10.2% in the insufficient EMS team group, respectively. After PSM, for the 968 patients, survival admission and discharge were 27.2% and 15.3%, respectively, in the sufficient EMS group, which is much better than that in the insufficient EMS group (19.4% and 8.3%, respectively) (Table 2).

**Figure 2:**
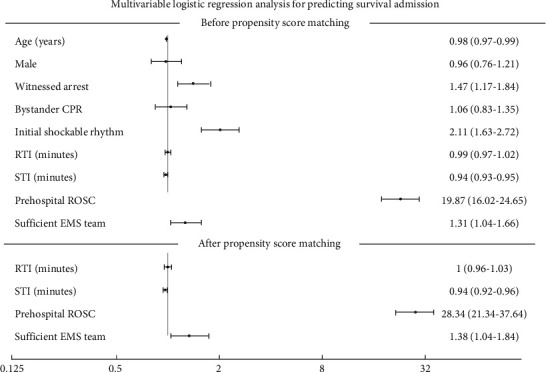
Forest plot of multivariable logistic regression analysis for predicting survival admission. Adjusted odds ratios for survival outcomes before and after propensity score matching with 95% confidence interval (CI). CPR, cardiopulmonary resuscitation; RTI, response time interval; STI, scene time interval; ROSC, return of spontaneous circulation.

In Table 3, multivariable analysis after PSM cohort (968 patients), adjusted survival to admission and discharge was significantly higher in the sufficient EMS team group than in the insufficient EMS team group: OR 1.37 (1.11–1.68) and 1.34 (1.03–1.74), respectively. After PSM, we demonstrated that STI (OR: 0.94) and prehospital ROSC (OR: 28.34) were independently associated with survival admission and discharge. Our results could not find a sufficient EMS team related to a good neurologic outcome (*P* value = 0.58) (Figure 2).

## 4. Discussion

Our study showed that the survival discharge of OHCA patients was higher in the six paramedics team than in the four or five paramedics team (15.3% vs. 11.9%) after PSM; moreover, it was similar to survival admission (27.1% vs. 21.4%) after PSM. However, there was no statistically significant difference in good neurologic outcomes at discharge after PSM in both groups (*P*=0.930).

We considered several factors that affect survival rate: age, sex, witnessed arrest, bystander CPR, initial shockable rhythm, RTI, STI, prehospital ROSC, and the number of EMS crew members [11, 12]. Our findings showed that age, witnessed arrest, initial shockable rhythm, STI, and prehospital ROSC had significantly different outcomes. In addition, before PSM, the sufficient EMS group showed a significant difference in survival until admission.

In meta-analysis, a pooled odds ratio for survival to discharge in the prehospital ROSC was 20.96–99.84 [13]. In our study, the odds ratio for survival to discharge in prehospital ROSC was 29.80, which is low compared to other studies. Since odds ratio depends on the variables included in the statistical analysis, direct comparison is inappropriate. However, there might be differences in in-hospital treatment depending on whether TTM was treated and the size of the hospital. Also, transfer to another hospital after in-hospital ROSC may adversely affect the patient's prognosis and these may affect the odds ratio of prehospital ROSC.

It is still controversial whether the number of EMTs improves the outcomes of OHCAs [14–16]. Previous studies have shown that the size of the crew of the prehospital ALS influences the outcome of cardiac arrest patients. According to Warren et al., the more the personnel involved in OHCA at the scene, the better is the survival discharge rate [5]. However, this study showed no significant difference in survival between five or six personnel versus fewer. According to a study conducted in the United States, 5-6 EMS personnel dispatched on scene accounted for 43.45% of the total cases, 22.64% for 7-8 personnel, and 5.94% for more than 8 personnel at the scene. On the other hand, it is rare in Korea to see more than seven EMS personnel dispatched on the scene; hence, we could not compare the outcomes for more than seven EMS personnel. Nevertheless, we derived the same tendency as Warren et al.; a more qualified CPR could be performed with more personnel on the scene.

A simulation performed by Tasai et al. showed that teamwork performance evaluated with the scoring of leadership dedication, rhythm management, medication communications, and chest compression fraction monitoring was better in five paramedics than six paramedics due to overcrowding in a confined space. However, the intervention time shortened as the crew size increased during manual CPR; hands-off time, time to the first dose of epinephrine, and time to complete the intubation were shorter than when performed with fewer personnel in this simulation CPR [6]. The actual situation in the OHCA field is different, and hence, the paramedics could not show the best performance; thus, this may have yielded different results in terms of teamwork performance.

Unlike these studies, Eschmann et al. investigated the effects on ROSC, survival till admission, and survival till discharge by dividing the ALS ambulance into two, three, and four or more. As a result, the increase in the number of paramedics was not associated with better outcomes. The reason for these results is that, first, the technique of ALS may not directly affect the patients and, second, the possibility of increased hands-off time during the ALS technique [7]. In our study, prehospital ALS was performed by completing regular courses under medical guidance and surveillance of the site in real time by a certified emergency physician. Therefore, the quality of direct medical guidance was better and the higher the number of paramedics implementing field ALS, the higher the survival rate.

### 4.1. Limitations

Our study has potential limitations. First, this was a retrospective study, and thus, there was no detailed information about prehospital ALS, hands-off time, intubation time, and time to the dose of first epinephrine. Thus, there was a significant loss of data regarding targeted therapeutic management. In addition, there was a lack of precise information on the EMS teams' level of technical ability. Second, the population of this study was from 12 different cities in the urban areas of Gyeonggi Province, Republic of Korea. We speculate that each city had some differences in the quality or size of the hospital. Therefore, the findings of this study cannot be generalized to other regions. Moreover, the OHCA patients were transferred to different hospitals and the patients' prognosis might vary depending on the hospital level and other circumstances [17]. Third, many cardiac arrests are not included, including CPR rejection, which could alter the result. Finally, in South Korea, since termination of resuscitation is not possible on-site unless the guardian refuses or there is an apparent death, the percentage of prehospital ROSC, survival to admission, survival to discharge, and good neurological outcome may seem relatively low. Also, in our study, only some equipped paramedics used a mechanical device during transport, which may have affected the patients' prognosis.

## 5. Conclusions

Our findings suggest that a sufficient EMS team (six personnel) could improve the survival till admission and the survival at the discharge of OHCA patients in prehospital ALS than an insufficient EMS team (four or five personnel).

## Figures and Tables

**Table 1:** Baseline characteristics of patients according to the EMS team personnel before and after propensity score matching.

	Before propensity score matching^a^			After propensity score matching^b^		
	Insufficient EMS team (*N* = 2,664)	Sufficient EMS team (*N* = 968)	SMD	Insufficient EMS team (*N* = 968)	Sufficient EMS team (*N* = 968)	SMD^c^
Age (years)	68.0 (55.0–79.0)	68.5 (55.0–79.0)	0.02	66.0 ± 15.4	66.0 ± 15.7	0.02
Male	1772 (66.5)	656 (67.8)	0.03	648 (66.9)	656 (67.8)	0.03
Witnessed arrest	1130 (42.4)	385 (39.8)	−0.05	382 (39.5)	385 (39.8)	−0.34
Bystander CPR	1944 (73.0)	650 (67.1)	−0.12	653 (67.5)	650 (67.1)	<−0.01
Initial shockable rhythm	520 (19.5)	204 (21.1)	0.04	191 (19.7)	204 (21.1)	−0.02

EMS, emergency medical services; SMD, standardized mean difference; CPR, cardiopulmonary resuscitation. ^a^Data before propensity score matching are expressed as median (interquartile range) or *N* (%) as appropriate. ^b^Data after propensity score matching are expressed as mean ± standard deviation or *N* (%) as appropriate. ^c^The SMD was calculated to compare variables after the propensity score matching. Imbalance was defined as SMD >0.10.

**Table 2:** Outcomes before and after propensity score matching.

	Before propensity score matching			After propensity score matching		
	Insufficient EMS team (4-5 of EMS) (*N* = 2,664)	Sufficient EMS team (6 of EMS) (*N* = 968)	*P*	Insufficient EMS team (4-5 of EMS) (*N* = 968)	Sufficient EMS team (6 of EMS) (*N* = 968)	*P*
Survival admission	540 (20.3)	263 (27.2)	0.03	188 (19.4)	263 (27.2)	<0.01
Survival discharge	272 (10.2)	148 (15.3)	0.06	80 (8.3)	148 (15.3)	0.03
Good neurologic outcome	169 (6.3)	72 (7.4)	0.13	53 (5.5)	72 (7.4)	0.93

EMS, emergency medical services.

**Table 3:** Multivariable logistic regression analyses of the variables associated with outcomes before and after propensity score matching.

Before propensity score matching									
	Survival admission			Survival discharge			Good neurologic outcome		
	Odds ratio	95% confidence interval	*P*	Odds ratio	95% confidence interval	*P*	Odds ratio	95% confidence interval	*P*
Age	0.98	0.97–0.99	<0.01	0.99	0.98–1.00	<0.01	0.97	0.95–0.98	<0.01
Male	0.96	0.76–1.21	0.75	1.21	0.89–1.65	0.23	1.16	0.73–1.84	0.54
Witnessed arrest	1.47	1.17–1.84	<0.01	1.59	1.19–2.13	<0.01	2.21	1.45–3.36	<0.01
Bystander CPR	1.06	0.83–1.35	0.65	1.18	0.85–1.62	0.32	1.55	0.97–2.47	0.07
Initial shockable rhythm	2.11	1.63–2.72	<0.01	3.57	2.67–4.77	<0.01	9.85	6.44–15.05	<0.01
RTI (minutes)	0.99	0.97–1.02	0.55	0.98	0.95–1.02	0.30	0.99	0.94–1.04	0.68
STI (minutes)	0.94	0.93–0.95	<0.01	0.92	0.91–0.94	<0.01	0.90	0.87–0.92	<0.01
Prehospital ROSC	19.87	16.02–24.65	<0.01	19.18	14.03–26.22	<0.01	27.86	15.38–50.45	<0.01
Sufficient EMS team	1.31	1.04–1.66	0.03	1.33	0.99–1.77	0.06	0.73	0.49–1.10	0.13
After propensity score matching									
	Survival admission			Survival discharge			Good neurologic outcome		
	Odds ratio	95% confidence interval	*P*	Odds ratio	95% confidence interval	*P*	Odds ratio	95% confidence interval	*P*
RTI (minutes)	1.00	0.96–1.03	0.76	0.98	0.93–1.02	0.28	0.97	0.92–1.03	0.34
STI (minutes)	0.94	0.92–0.96	<0.01	0.92	0.90–0.94	<0.01	0.88	0.85–0.91	<0.01
Prehospital ROSC	28.34	21.34–37.64	<0.01	29.80	19.66–45.17	<0.01	56.12	25.77–122.21	<0.01
Sufficient EMS team	1.38	1.04–1.84	0.03	1.70	1.20–2.40	0.03	0.88	0.57–1.36	0.58

EMS, emergency medical services; SMD, standardized mean difference; CPR, cardiopulmonary resuscitation; RTI, response time interval; STI, scene time interval; ROSC, return of spontaneous circulation.

## Data Availability

No data were used to support this study.
